# Cultural models within general practice/family medicine training: a scoping review protocol

**DOI:** 10.1136/bmjopen-2025-099361

**Published:** 2025-08-27

**Authors:** Lisa Collins, Helen Reid, Hinemoa Elder, Grainne Kearney

**Affiliations:** 1 Dentistry and Biomedical Sciences, Queen’s University Belfast, Belfast, UK; 2 School of Graduate Studies, Te Whare Wānanga o Awanuiārangi, Auckland, New Zealand; 3 Te Hiku Hauora, Kaitaia, Aotearoa, New Zealand

**Keywords:** Primary Care, MEDICAL EDUCATION & TRAINING, MEDICAL ETHICS

## Abstract

**Abstract:**

**Introduction:**

Cultural competency, cultural safety, cultural humility and transcultural care have developed as frameworks to better equip medical professionals towards a more culturally appropriate healthcare system. The aim of this scoping review is to map the use of these cultural models within general practice/general practitioner (GP) training. We have elected to use the term ‘GP’ to encompass all trainee doctors within general practice/family medicine.

**Methods and analysis:**

This scoping review will be conducted in accordance with the Scoping Review Methods Manual by the Joanna Briggs Institute and the Arksey and O’Malley framework for scoping studies and reported using the Preferred Reporting Items for Systematic Reviews and Meta-Analyses extension for Scoping Reviews. Searches were conducted in EMBASE, MEDLINE and Web of Science Core Collection, with the support of a subject librarian. Published literature on cultural competence, cultural safety, cultural humility and transcultural care related to GP training will be included. There will be no restriction placed on language. References will be managed on EndNote, and titles and abstracts will be screened against the inclusion criteria by two independent reviewers. Potentially relevant sources will be retrieved in full and their citation imported into Rayyan. Data will be extracted on the year, type of study, country or countries of affiliated authors, characteristics of participants, research design and setting, cultural model being examined, definitions used, attitudes, outcome and application of the model, and purpose of the study. We aim to use basic qualitative content analysis for data extracted to map the landscape of the published literature around cultural competence, cultural humility, cultural safety and transcultural care.

**Ethics and dissemination:**

Ethics approval was not required for this Scoping Review protocol. Findings will be disseminated through conference presentations and publication in a scientific journal.

**STRENGTHS AND LIMITATIONS OF THIS STUDY**

- This review will entail a rigorous search on multiple databases for the cultural models of cultural competence, cultural safety, cultural humility and transcultural care as relevant to GP training.
- The methodology follows Preferred Reporting Items for Systematic reviews and Meta-Analyses extension for Scoping Reviews and Joanna Briggs Institute guidelines, ensuring transparency and replicability.
- All articles that meet the inclusion criteria, regardless of language, will be included in our study.
- Due to feasibility, our database search will be limited to the English terms of ‘cultural competence’, ‘cultural safety’, ‘cultural humility’ and ‘transcultural care’. Other cultural models may exist but not be found and explored during this review due to capacity.

## Introduction

 Evidence shows that shared decision-making regarding treatment and understanding between general practitioners (GPs) and their patients can be problematic in cross-cultural consultations.^1^,^3^ Teunissen *et al* explain cross-cultural communication within GP to mean when ‘patients and doctors do not share language or culture’, highlighting that ‘basic communication can become problematic, with detrimental effects on access, outcomes and safety’.^1^ GPs are increasingly working in cross-cultural consultations, so it is vital that their care is culturally appropriate. A patient’s culture is intrinsic to who they are, the beliefs they hold and the decisions they make. As healthcare professionals, culture impacts the care delivered. Recognising and acknowledging how a person’s culture influences disease, health and treatment has been identified as central to deliver effective healthcare.^4^

GPs act as gatekeepers for patients accessing healthcare, especially if further care is needed within a secondary care setting. Presenting to a GP is often the first step that people take when seeking healthcare in a new country. Starfield concluded that inequity is built into health systems, but primary care can offer ‘preferential benefit to the socially disadvantaged’.^5^ Acknowledging the role of cultural awareness and its potential impact on reducing health inequities is an ongoing area of research.^6^

Language barriers, time constraints, interpreter use and lack of familiarity with cultural belief systems have all been reported as examples of the challenges faced by GPs.^7^,^9^ Miscommunication and mistrust result if cultural differences between patients and professionals are not addressed.^10^ GPs have stated their concerns around inadequate training to serve the needs of a diversifying population.^8^

### Cultural models

Cultural model frameworks have developed and been implemented as a means of better serving all patients and providing culturally specific care.^11^,^15^ Cultural competency, cultural safety, cultural humility and transcultural care have evolved as models to better equip medical professionals, their patients and health systems, in their use of language and clinical management, striving towards a more culturally appropriate healthcare system and consequently ensuring equity and culturally meaningful outcomes for patients. Few studies have focused on the training of doctors in cultural models.^14^

Cultural competency, perhaps best known, is a broad model that advocates for patient-centred care^12 16^ with skills, knowledge and attitudes necessary to provide quality care that aligns with the patient’s cultural health and belief practices, which can reduce health inequities.^17^,^23^ Training in culturally competent care remains underdeveloped in GP curricula despite GP trainees expressing a desire to be better trained as culturally competent healthcare practitioners.^14 24 25^

There has been much reported on what constitutes cultural competence and debate surrounding where the framework originated.^16^ Leininger argued that the phrase was first used by her in the 1960s as part of a theory of cultural care diversity.^26^ Cross *et al* can be found making use of the term in 1989, defining cultural competence as ‘a set of congruent behaviours, attitudes and policies that come together in a system, agency or among professionals and enable that system, agency or those professionals to work effectively in cross-cultural situations’.^27^ DeSantis referenced cultural competence in terms of nursing in the 1990s.^28^

Seeleman *et al* in 2009 set out three domains of cultural competency, including ‘knowledge’, which encompasses the epidemiology and the impact of treatment in different ethnic groups; ‘attitudes’, an awareness of how culture shapes thinking and behaviour and ‘skills’, an ability to convey information in a manner that the patient understands.^29^ Fernandez *et al* found that language and cultural competence skills make a significant difference in health communication.^30^ There is evidence that interventions to improve cultural competency can improve patient outcomes, but further research is needed.^12 31^

Criticism of cultural competence has focused on its failure to examine the inherent power dynamic between the patient and doctor and how healthcare professionals can never be truly ‘competent’ in understanding another person’s culture.^32^,^34^ It has also been argued that the term cultural competence is too often used interchangeably with other models and that this has the potential to confuse medical professionals. Lokugamage *et al* propose more consistent use of the term ‘cultural safety’ as it acknowledges inherent power imbalances between doctor and patient.^35^

Cultural safety originated in New Zealand in response to structured racism experienced by the Māori population.^11 36 37^ Coined by Māori nurse Irahapeti Ramsden, cultural safety places a focus on the healthcare professional and the impact that their own cultural systems can have on the doctor–patient relationship. Originally, Ramsden argued that cultural safety required the development of cultural awareness to cultural sensitivity with progression to cultural safety.^11^ Belfrage, who dedicated her career to working with indigenous communities in Central Australia, argued that engaging with cultural safety can ensure that people believe that healthcare is connected to their lives, that they are involved in choices, where it is ‘not so much about empowering people as not disempowering’.^38^ Cultural safety continues to evolve with Lokugamage *et al* in 2021 identifying that cultural safety aims to ‘dismantle barriers faced by colonised Indigenous people in mainstream healthcare by addressing systemic racism’.^35^ Mashford-Pringle *et al,*^39^ in their COVID-19 analysis with Indigenous populations, state that cultural safety has developed as a continuum moving from cultural awareness to cultural sensitivity to cultural competence and finally cultural safety. They also remind us that a key aspect of cultural safety is the self-determination of those receiving care.^39^

Cultural humility was developed in 1998 to address the perceived limitations of cultural competence.^40^ It has been revered as the model to eliminate power imbalances, as ‘a process of openness, self-awareness, being egoless and incorporating self-reflection and critique after willingly interacting with diverse individuals’.^41^ The overlap between cultural safety and cultural humility has been acknowledged, with the difference that cultural safety focuses on the self-reflection of internalised prejudices, whereas cultural humility tends to externalise to focus on the experiences of the patient with lifelong learning and acknowledgement of our own inherent biases.^42 43^ That more consistent definitions are needed for all models is echoed throughout the literature.^13 44 45^

There are no studies that map the application of these cultural models, how they are applied, attitudes towards them, and their prevalence and training within GP and family medicine training schemes worldwide.^14^ We have elected to use the term ‘GP’ to encompass all trainee doctors within general practice/family medicine. These doctors are professionals, often referred to as registrars or trainees, who post medical school have completed mandatory hospital jobs and have elected to join general practice training schemes.^46^

### Objectives

The primary objective of this scoping review is to map and summarise existing research available in peer-reviewed and grey literature on the models of cultural competence, cultural safety, cultural humility and transcultural care within GP training worldwide. A secondary objective is to explore how the models are applied within GP training and attitudes surrounding them. Findings from this review will offer a greater understanding of cultural models within the specific context of GP training schemes, and summarising recurring themes will help identify gaps in knowledge to guide future research and service provision. We additionally hope that by mapping the use of cultural models within GP training schemes, this will inform future research plans.

## Methods and analysis

To ensure rigour and replicability of our proposed scoping review, it will be conducted in accordance with the Joanna Briggs Institute (JBI) updated methodological guidance.^47^ This draws on the framework of Arksey and O’Malley, and our scoping review is therefore conducted using the five stages of this framework: (1) identifying a research question, (2) identifying relevant studies, (3) study selection, (4) charting the data and (5) collating, summarising and reporting results.^48^ The results of the search and the study inclusion process will be reported in full in the final scoping review and presented using the guidelines from the Preferred Reporting Items for Systematic reviews and Meta-Analyses extension for Scoping reviews (PRISMA-ScR).^49^ This will demonstrate clearly where potential studies/sources were excluded during the screening process. A rationale for the exclusion of sources during full-text screening will also be provided.

A preliminary search of EMBASE, MEDLINE and Web of Science Core Collection was conducted, and no current or underway scoping reviews on the topic were identified. We anticipate completion in 2025.

### Stage 1: identify the research question

In consultation with our research team, we developed a broad research question: ‘how are the cultural models of cultural competency, cultural safety, cultural humility and transcultural care incorporated and explored within GP training worldwide?’. Levac *et al* encourage researchers to define target populations in scoping reviews.^50^ For this review, we have elected to use the term ‘GP’ to encompass all trainee doctors within general practice/family medicine training schemes worldwide.

### Stage 2: identifying relevant studies

#### Inclusion criteria

To develop the eligibility criteria, the participants, concept and context framework was used, and these criteria were developed in accordance with the study objectives (table 1).^51^

**Table 1:** Study participants, concept, context and types of evidence

	Inclusion	Exclusion
Participants	Human participants who are doctors working as GP trainees, including those identified as family medicine/practice residents, to incorporate from all countries worldwide.	Studies that do not include human participants.
Concept	Any study with a primary objective to define, discuss or explore the cultural models of ‘cultural competence’, ‘cultural safety’, ‘cultural humility’ and ‘transcultural care’ within GP training worldwide.	Studies pertaining to GP and not GP training. Studies in other medical disciplines outside GP training. Additional cultural models that are not cultural competency, cultural humility, cultural safety and transcultural care.
Context	Any geographical location.	None.
Types of evidence	All published and grey literature. No limitation on the year of publication. All languages.	Editorial articles. Oral presentations and posters. Blogs.

GP, general practitioner.

#### Search strategy

The initial search strategy was developed using an iterative approach,^52^ with two rounds of preliminary searches and refinement of the search strategy based on results retrieved. A pilot search was undertaken on Google Advanced Search in May 2023. We were particularly keen to identify examples of cultural models that could be included in our review, and initially we identified the terms ‘cultural competence’, ‘cultural safety’ and ‘cultural humility’. At this point, a subject librarian was consulted to build a transparent and robustly structured search strategy. From this initial search, we also identified an additional cultural model term, ‘transcultural care’, and incorporated this into our next iteration of the search strategy. The preliminary search strategy aimed to locate both published and unpublished studies. An initial literature search was done in EMBASE, MEDLINE and Web of Science Core Collection to identify articles on the topic. These databases were selected by the experienced librarian. Boolean operators OR and AND were used. The text words contained in the titles and abstracts of relevant articles, and the index terms used to describe the articles, were used to develop a full search strategy. After a brief review of the preliminary results, we ascertained that using only ‘general practice/GP’ wasn’t capturing the full scope of doctors working within this setting and thus included the term ‘family medicine’ and repeated the search.

A second preliminary search was conducted in July 2023. Rayyan, an Artificial Intelligence-powered tool for collating literature reviews,^53^ detected and removed duplicates. The search was rerun in September 2024 to identify any new or additional sources that may be relevant for the scoping review. At this point, we have decided to include ‘all languages’ in our search, as previously we had only included those that were in English. We felt this would be more relevant and inclusive for our study and ensured that we were not missing important articles. We will supplement this search by exploring the reference lists of selected articles retrieved to identify any additional studies that may not have been captured in the database results. We will also be exploring any grey literature, such as curricula and dissertations. The results returned from each database can be found in online supplemental appendix S1.

### Stage 3: study selection

References will be managed with EndNote software. Non-English articles will be translated using Google Translate, which has been used in previous reviews.^54^ Titles and abstracts will be screened against the inclusion criteria by two independent reviewers. Articles will be excluded if the topics or outcomes are deemed irrelevant to the scope of our study. Potentially relevant sources will be retrieved in full and their citation details imported into Rayyan. Any disagreements that arise between the reviewers at each stage of the selection process will be resolved through discussion or with an additional reviewer until consensus is reached. If sources are excluded, this will be reported in the scoping review.

Whenever a potentially relevant resource is not readily available, or access is restricted, the relevant authors will be contacted to request a copy.

### Stage 4: charting the data

Data will be extracted by the primary author, who will also perform the screening and full-text reviews to ensure consistency, which will be shared with other authors. Two reviewers will regularly meet to discuss the findings of the extracted data, including key findings and themes. We plan to extract information on the type of study, country or countries of affiliated authors, characteristics of participants, research design and setting, cultural model being examined, aim/outcome of the study, design and intervention, and purpose and key findings (online supplemental table 1). Under these main headings, we will also extract information on how the cultural model has been defined, if at all, attitudes towards the model and how they have been applied within training schemes. A modified data extraction table, as set out by the JBI, will be used for data collection.^46^ Each paper will be assigned an identification number, and findings related to the research question will be summarised and coded. The data extraction table will be modified, if necessary, during the extraction process and, if required, this will be detailed in the full scoping review.

To present the findings across studies, as a team, we will engage in coding and the development of themes linked to the research question and objectives. As is common in scoping reviews, information will be analysed qualitatively rather than quantitatively.^55^ We aim to follow the JBI updated methodological scoping review guidance recommendation of using basic qualitative content analysis^47^ for data extraction. As per Pollock *et al,*^52^ we will also be guided by Elo and Kyngas’ three phases of qualitative content analysis: preparation, organising and reporting.^56^ We will use an inductive approach for extraction and analysis.

The review team will provide a descriptive analysis of the evidence collated. The PRISMA-ScR checklist will be used to ensure methodological rigour and enhance the reporting of the results.^49^ We will not report on the quality of evidence or synthesise findings in line with JBI methodology.^47^

Best practice for scoping reviews recommends a team approach. We aim to meet regularly as a team throughout the review process, including during data extraction and analysis.^52^

### Stage 5: collating, summarising and reporting the results

We intend to follow the suggestions from Levac *et al* in terms of how this stage of the scoping review is divided: analysing the data, reporting results and applying meaning to the results in terms of implications for future research and practice.^50^ Key findings and themes will be communicated through tables, figures, oral presentations and peer-reviewed publications. In reporting our results, we will highlight similarities and differences in how cultural models are used.

### Ethics and dissemination

Ethics approval was not required for this scoping review protocol, as we have no intention to collect primary data. As such, approval from a research ethics committee is not required. Findings of the scoping review will be disseminated through conference presentations and publication in a scientific journal.

### Patient and public involvement

This scoping review analyses existing research studies and thus does not involve patients or members of the public.

### Implications

Cultural competence, cultural humility, cultural safety and transcultural care have developed as models to support culturally appropriate care. This scoping review aims to provide an overview of how these cultural models are being used within GP and family medicine training around the world and identify any gaps that exist in the literature. By doing this, we can explore knowledge and attitudes towards the models, how this learning, if at all, has been incorporated into GP training, who is delivering the training and the attitudes towards it, resulting in healthcare that offers more culturally appropriate care and the potential to better serve all patients.

### Strengths and limitations

Strengths: this review will entail a rigorous search for the cultural models of cultural competence, cultural safety, cultural humility and transcultural care as relevant to GP training. A number of researchers will be involved in data collection and analysis, enhancing the quality of the review produced. All articles that meet the inclusion criteria, regardless of language, will be included in our study.

Limitations: due to feasibility, our database search will be limited to the English terms of ‘cultural competence’, ‘cultural safety’, ‘cultural humility’ and ‘transcultural care’. Due to capacity, we will not be able to search for these terms in alternative languages. There is also the risk of inaccuracies with translating any non-English articles into English via Google Translate, and information may be lost in the translation process. Furthermore, other cultural models may exist but not be found and explored during this review due to capacity.

## Supplementary material

10.1136/bmjopen-2025-099361 online supplemental file 1
