# Supplementary material for: Cultural models within general practice/family medicine training: a scoping review protocol
Source: BMJ Open. 2025 Aug 27;15(8):e099361. doi: 10.1136/bmjopen-2025-099361 (PMC12410604; doi:10.1136/bmjopen-2025-099361)
Supplement: online supplemental file 1 [file bmjopen-15-8-s001.docx]

**Supplementary Appendix S1.**

Search Strategy for MEDLINE, EMBASE and Web of Science.

Web of Science:


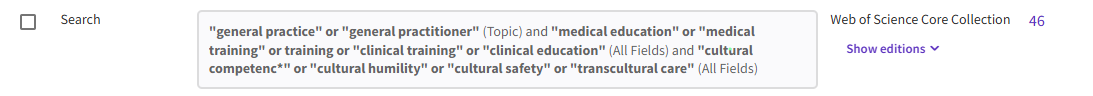


Medline:


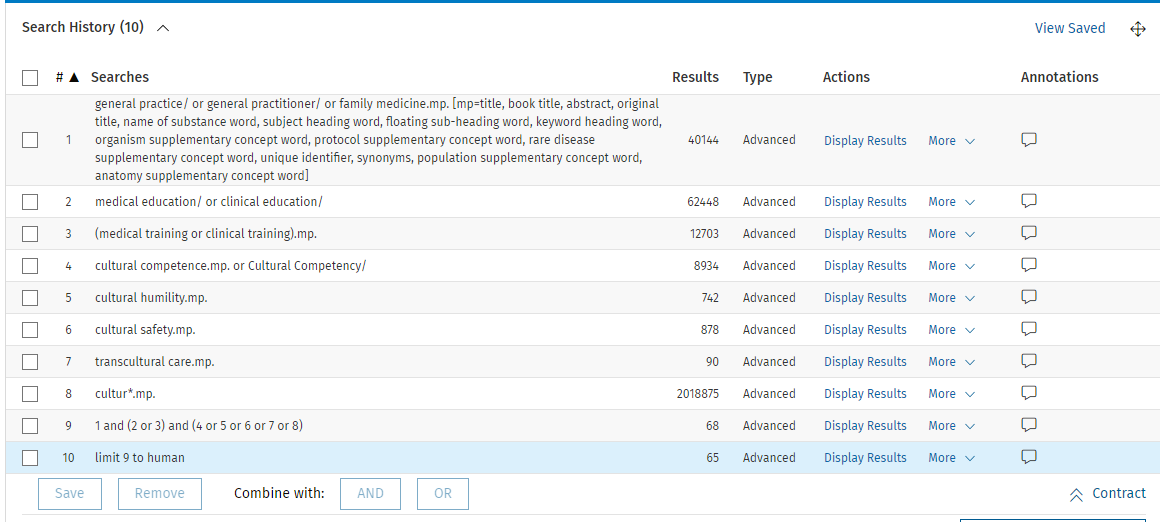


Embase:


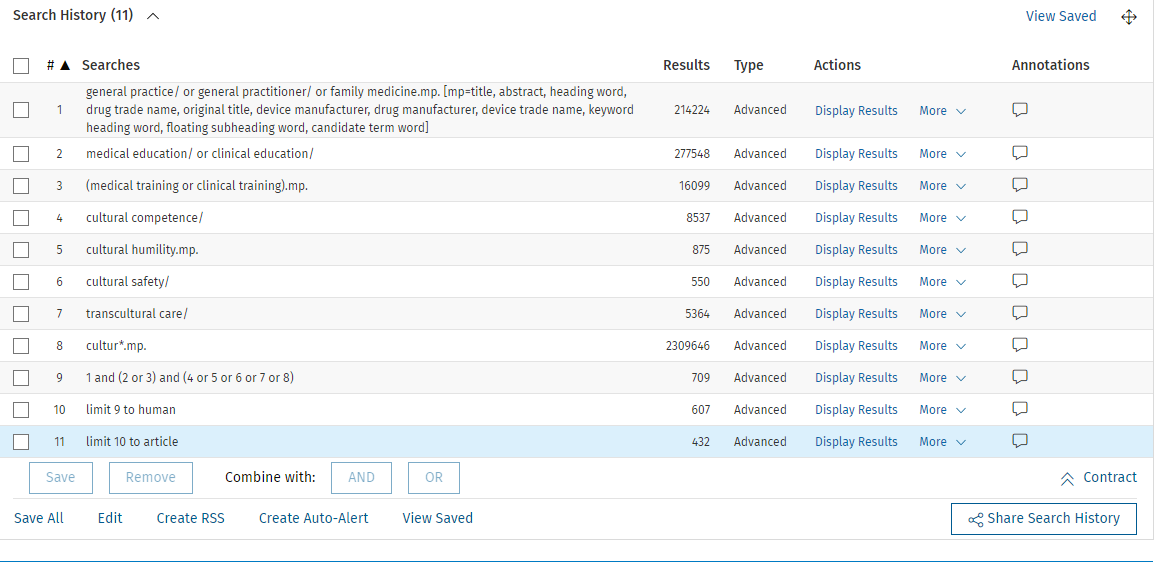


**Supplementary Table 1**: Included Articles for Scoping Review.

|  | **No.** | **Author(s) & Year** | **Title** | **Country/ Context** | **Participants** | **Cultural model** | **Aim** | **Design/Intervention** | **Summary** | **Purpose/Key Findings** | **Themes/Coding** |
| --- | --- | --- | --- | --- | --- | --- | --- | --- | --- | --- | --- |
