# Supplementary material for: Cultural models within general practice/family medicine training: a scoping review protocol
Source: BMJ Open. 2025 Aug 27;15(8):e099361. doi: 10.1136/bmjopen-2025-099361 (PMC12410604; doi:10.1136/bmjopen-2025-099361)
Supplement: Supplementary file 2 [file bmjopen-2025-099361.reviewer_comments.pdf]

PEER REVIEW HISTORY

BMJ Open publishes all reviews undertaken for accepted manuscripts. Reviewers are asked to complete a checklist review form and are provided with free text boxes to elaborate on their assessment. These free text comments are reproduced below.

ARTICLE DETAILS

Title (Provisional)

Cultural Models within General Practice/ Family Medicine Training: a Scoping Review Protocol

Authors

Collins, Lisa; Reid, Helen; Kearney, Grainne; Elder, Hinemoa

VERSION 1 - REVIEW

**Reviewer**	**1**
**Name**	**Zaccagnini , Marco**
**Affiliation**	**McGill University**
**Date**	**18-Apr-2025**
**COI**	**None**

Thank you for letting me review “Cultural Models within General Practice/ Family Medicine Training: a Scoping Review Protocol” for BMJ Open. Please take my comments in the spirit that they were written, to improve the content in your paper. Overall the paper is well written but there are locations that might be strengthened, I include detailed notes in the PDF. I will highlight some of the more prominent issues.

The section about debate about the framework of cultural competence is quite long, I would suggest giving a brief and succinct outline instead of referencing all of the key researchers that have discussed this topic. Furthermore, you start with a discussion of cultural competence and a debate about where the framework started, then move into a section about domains, followed by evidence of training in cultural competency. There are sentences missing in between these concepts to bridge your story together.

One of your objectives is to make recommendations. However, given that scoping reviews are inherently exploratory, they should not be used to make recommendations. This language should be tempered. Please see the following authoritative papers:

Peters, M.D.J., Marnie, C., Colquhoun, H. *et al.* Scoping reviews: reinforcing and advancing the methodology and application. *Syst Rev* **10**, 263 (2021). https://doi.org/10.1186/s13643-021-01821-3

Pollock D, Davies EL, Peters MDJ, Tricco AC, Alexander L, McInerney P, Godfrey CM, Khalil H, Munn Z. Undertaking a scoping review: A practical guide for nursing and midwifery students, clinicians, researchers, and academics. J Adv Nurs. 2021 Apr;77(4):2102-2113. doi: 10.1111/jan.14743.

In the methodology, there is no research question, Stage 2 (search strategy) has aspects of results interwoven in it and the grey literature search is not outlined.

Finally, there is not a sufficient analytic plan for the extracted information. There are multiple ways to analyze the data (e.g., qualitative content analysis, reflexive thematic analysis), however, details should be provided for the reader. See: Pollock D, Peters MDJ, Khalil H, McInerney P, Alexander L, Tricco AC, Evans C, de Moraes ÉB, Godfrey CM, Pieper D, Saran A, Stern C, Munn Z. Recommendations for the extraction, analysis, and presentation of results in scoping reviews. JBI Evid Synth. 2023 Mar 1;21(3):520-532. doi: 10.11124/JBIES-22-00123

**** **The reviewer provided a marked copy with additional comments. Please contact the publisher for full details.** ****

**Reviewer**	**2**
**Name**	**Brumpton, Kay**
**Affiliation**	**Griffith University**
**Date**	**12-May-2025**
**COI**	**None**

Thank-you for your work in developing this scoping review. The review is timely and appropriate.

There are several concerns that I feel need to be addressed for this work to be published.

1. I suggest the review is either narrowed to focus on refugee/displaced peoples' health or the introduction broadened to be inclusive of other cultural or intersectional contexts. Currently the paper presents UK displaced people and Indigenous Australians without detailing the difference in contexts.

2. Much of the evidence provided is from >20 years ago. The manuscript should be revised to provide more recent evidence. The evidence in this space has progressed significantly within this time.

3. More clearing defining what is meant by cultural models and consistency in language around these. For example, are a cultural model and a tool the same?

4. The current objective - 'to bring together research, map evidence and their presence" - does not address the gap identified in the introduction regarding how these models are

applied, and attitudes towards them. Page 6 line 48 clarification is required around "and the disparities that exist between different training schemes".

5. To be able to replicate the study it is important to know how GP training is defined. For example, is this from medical student through to continuous professional development or other? Why was the term 'primary care' or 'family physician' or other terms not included?

6. Grey literature is included but there are no details on how the grey literature will be searched. Furthermore, limitations indicate that perhaps most grey literature is excluded?

7. Data extraction – this study would benefit from reviewing additional data fields such as the definitions uses, application of model, outcomes, attitudes etc.

8. The final sentence of the manuscript (p11 line 3) would benefit from an additional line to describe the "so what / why" this is important.

9. The strengths and limitations need to consider the authors reflexivity, further clarification of how rigour is determined, including the number of researchers.

10. General:

- GP needs to be checked for consistency: general practice and general practitioner are not interchangeable.

- Check all statements are appropriately referenced.

- Pg 4 line 10-14 a systemic review is attributed to two references.

- Pg 4 Sentence starting Line 17 would benefit from rewording

- References need to be checked for grammar, applying the et al. rule consistently, year provided where possible, completeness (for example 28)

VERSION 1 - AUTHOR RESPONSE

Reviewer 1: Mr. Marco Zaccagnini , McGill University

General Comments:

Thank you for letting me review "Cultural Models within General Practice/ Family Medicine Training: a Scoping Review Protocol" for BMJ Open. Please take my comments in the spirit that they were written, to improve the content in your paper. Overall the paper is well written but there are locations that might be strengthened, I include detailed notes in the PDF. I will highlight some of the more prominent issues

Response:

Thank you for your recognition of this important topic. We appreciate your interest in the paper and your time and efforts in reviewing our work.

	Issue raised in peer review	Response	Where addressed in revision
1.	The section about debate about the framework of cultural competence is quite long, I would suggest giving a brief and succinct outline instead of referencing all of the key researchers that have discussed this topic. Furthermore, you start with a discussion of cultural competence and a debate about where the framework started, then move into a section about domains, followed by evidence of training in cultural competency. There are sentences missing in between these concepts to bridge your story together	We appreciate your feedback on this area and have decided to revise the introduction based on these suggestions. The second reviewer also suggested a broader introduction with more up to date research referenced. We have attempted to encompass both of your suggestions.	Introduction revised to incorporate your suggestions, particularly trying to be more succinct when explaining the cultural models and referencing more relevant and updated research. Pages 2-5
2.	One of your objectives is to make recommendations. However, given that scoping reviews are inherently exploratory, they should not be used to make recommendations.	We appreciative your guidance around this and the papers that you provided.	We have deleted the word "recommendations" from the Objectives section and focused more on the primary objective of this scoping review, which is to map and summarise existing research available in peer-reviewed and grey literature on the models of cultural competence, cultural safety, cultural humility and transcultural care within GP training worldwide. We have outlined that this will permit evidence to be collated in a coherent manner and that findings from the review will offer greater understanding of cultural models within the specific context of GP training schemes.

			Summarising recurring themes will help identify gaps in knowledge to guide future research and service provision.<br><br>See Page 5.
3.	In the methodology, there is no research question, Stage 2 (search strategy) has aspects of results interwoven in it and the grey literature search is not outlined.	Thank you for your feedback here.	We have updated the research questions for the scoping review.<br>We have removed the detail on the number of articles retrieved from the initial searches and have added in that grey literature, such as curricula and phd dissertations, will also be analysed.<br><br>Pages 5-8.
4.	Finally, there is not a sufficient analytic plan for the extracted information. There are multiple ways to analyze the data (e.g., qualitative content analysis, reflexive thematic analysis), however, details should be provided for the reader	We appreciate your guidance on explaining better how we hope to analyse the extracted data.	Pages 8-9.<br><br>You will see that we have outlined how we plan to chart the extracted data. We have provided more specific detail on how we will use the JBI updated methodological scoping review guidance recommendations of analysis. We also found the paper you provided by Pollock et al. very useful and have included this.
5.	Page 4, L8: This is sentence sounds blunt. I would suggest finding a way to combine the two sentences so they flow better.	Thank you for this and we agreed this sentence needed amended. Having taken all feedback into consideration we have	Removed to make introduction more general.<br><br>Pages 2-3.

		decided to make the introduction more general.	
6.	Page 4, L54: I would avoid the word emerged because it's a misnomer. These frameworks don't emerge from the ground. They were developed and implemented as a means of better serving...	We agree with this suggestion. The frameworks have developed rather than emerged.	I have deleted the word "emerged" and included your suggestion of their development and implementation to better serve all patients. Page 3.
7.	Page 4, L55: I would expect to see some references of cultural model frameworks.	We agree that references were missing at this point in the discussion for the cultural frameworks.	Included. Page 3.
8.	Page 5, L7: This sentence is awkward.<br>Is cultural competency a model or a concept?	We feel that cultural competency is a model.	Revised to identify cultural competency as a model and not a concept (Page 3). Have made this consistent throughout the manuscript.
9.	Page 7, L21: You sometimes call it a stage and sometimes a step. Please be consistent.	As per your suggestion throughout the paper we have attempted to be more consistent with all terminology.	Have revised to refer only to the Stages of the Arksey O'Malley framework for scoping reviews. Page 5.
10.	Page 8, L3: You've included extra information that is likely not interested. Please just outline the population, the follow section is about the concept.	Thank you for this helpful feedback and we have amended our paper as per your suggestions.	Please see Table 1 outlining the Study Participants, Concept, Context and Types of Evidence. (Pages 6-7)
11.	Page 8, L16: Concept and context are two different aspects of the framework, they should be explained and mutually exclusive.	We appreciate your guidance on this and have amended appropriately.	As above Table 1. Pages 6-7.
12.	Page 10, L5: This is not a sufficient analytic plan for the extracted information. There are multiple ways to analyze the data (e.g., qualitative content analysis, reflexive thematic analysis), however, details should be provided for the reader.<br>See: Pollock D, Peters MDJ, Khalil H, McInerney P, Alexander L, Tricco AC, Evans	We agree that it is very important in a scoping review protocol to outline for the reader how extracted data will be analysed. We also appreciate the very	See pages 8-9 where we have clearly outlined how data will be charted and analysed.

	C, de Moraes ÉB, Godfrey CM, Pieper D, Saran A, Stern C, Munn Z. Recommendations for the extraction, analysis, and presentation of results in scoping reviews. JBI Evid Synth. 2023 Mar 1;21(3):520-532. doi: 10.11124/JBIES-22-00123	helpful paper by Pollock et al.	
13.	Page 10, L26: Thematic analysis is mentioned here, but should be included above with an analytic plan regarding how the authors will do it.	We have now moved this to the section above.	See pages 8-9.
14.	Page 11, L2: And knowing/mapping this will do what exactly? Why is this work needed?	Thank you for this comment. It is important for the reader to know exactly why we think this work is important/ needed.	Page 10. This scoping review aims to provide an overview of how these cultural models are being utilised within GP and family medicine training around the world and identify any gaps that exist in the literature. By doing this we can explore knowledge and attitudes towards the models, how this learning, if at all, has been incorporated into GP training, who is delivering the training and the attitudes towards them, resulting in healthcare that has the potential to better serve all patients and reduce health inequities.

Reviewer 2

Prof. Kay Brumpton, Griffith University, James Cook University

Comments to the Author:
Thank-you for your work in developing this scoping review. The review is timely and appropriate.

Response:

Thank you for your recognition and interest in our paper and the time you have taken to review and constructively critique our work.

	Issue raised in peer review	Response	Where addressed in revision
1.	I suggest the review is either narrowed to focus on refugee/displaced peoples' health or the introduction broadened to be inclusive of other cultural or intersectional contexts. Currently the paper presents UK displaced people and Indigenous Australians without detailing the difference in contexts.	We appreciate your feedback on this area and have decided to revise the introduction based on these suggestions. The first second reviewer also suggested a more succinct outline of the cultural models. We have attempted to encompass both of your suggestions.	Introduction revised to incorporate your suggestions, particularly trying to broaden the context and referencing more relevant and updated research. Pages 2-5
2.	Much of the evidence provided is from >20 years ago. The manuscript should be revised to provide more recent evidence. The evidence in this space has progressed significantly within this time.	Thank you for your feedback on the references. We agree that research on the area has progressed significantly.	References updated to reflect more recent evidence. Pages 2-5
3.	More clearing defining what is meant by cultural models and consistency in language around these. For example, are a cultural model and a tool the same?	Thank you for this. Throughout the paper we have attempted to be more consistent with language used.	Have revised paper to only refer to models and not tools. Page 3.
4.	The current objective - 'to bring together research, map evidence and their presence" -does not address the gap identified in the introduction regarding how these models are applied, and attitudes towards them. Page 6 line 48 clarification is required around "and the disparities that exist between different training schemes".	We agree that the objectives of the review needed to be more clearly articulated and	Clarified and updated in both introduction and objectives. The line "and the disparities

		aligned with the introduction.	that exist between different training schemes has been removed" Pages 2-3, 5.
5.	To be able to replicate the study it is important to know how GP training is defined. For example, is this from medical student through to continuous professional development or other? Why was the term 'primary care' or 'family physician' or other terms not included?	We have taken time to consider how we identify GP training and included this in the paper.<br><br>Thank you for your feedback on the use of 'Primary Care'. We deliberately choose not to include the term 'primary care' as we wanted to focus only on GP trainees and not other medical health professionals, which primary care tends to encompass.	1)We have identified more clearly what is meant by "general practitioner". (Introduction Page5 and Stage 1: Identify Research Question Page 6)<br><br>2)We have updated the supplementary information to include our search strategy for Web of Science Core Collection, Embase and Medline. You will see that this includes "family medicine". The search strategy was also conducted with a subject librarian who was confident that we would be capturing all relevant material. (See Supplementary Appendix S 1 Pages 7-8).

6.	Grey literature is included but there are no details on how the grey literature will be searched. Furthermore, limitations indicate that perhaps most grey literature is excluded?	We thank you for your feedback on this and we apologise our noted limitations did not match up the main body of the text. As part of our searches we examined GP training curricula and phd dissertations, referencing Ramsdens (2022) phd thesis on her work with the Māori population in New Zealand and cultural safety.	Curricula and PhD dissertations have been searched as grey literature. See included reference 11 for Ransden's (2002) seminal work on cultural safety. This has been detailed in the manuscript.<br><br>Pages 7-8.
7.	Data extraction – this study would benefit from reviewing additional data fields such as the definitions uses, application of model, outcomes, attitudes etc.	Thank you for this feedback. We have provided a copy of our data extraction table as supplementary information and also updated this in our paper. We have a number of main headings including: type of study, country or countries of affiliated authors, characteristics of participants,	See Page 8 and Supplementary Table 1.

		research design and setting, cultural model being examined, aim/outcome of the study, design and intervention, and purpose and key findings. (See Supplementary Table 1 and Page 8). Under these main headings we will also extract information on how the cultural model has been defined, if at all, attitudes towards the model and how they have been applied within training schemes. A modified data extraction table, as set out by the JBI will be utilised for data collection.	
8.	The final sentence of the manuscript (p11 line 3) would benefit from an additional line to describe the "so what / why" this is important.	Thank you for this feedback. We have updated the manuscript to more clearly outline why we think this work is important under the title 'Implications'.	Page 10. This scoping review aims to provide an overview of how these cultural models are being utilised within GP and family medicine training around the world and identify any gaps that exist

			in the literature. By doing this we can explore knowledge and attitudes towards the models, how this learning, if at all, has been incorporated into GP training, who is delivering the training and the attitudes towards them, resulting in healthcare that has the potential to better serve all patients and reduce health inequities.
9.	GP needs to be checked for consistency: general practice and general practitioner are not interchangeable.	We have attempted to be more consistent with terminology throughout the manuscript and appreciate your feedback on this.	Updated throughout the manuscript.
10.	Pg 4 line 10-14 a systemic review is attributed to two references.	Apologies for this oversight.	This reference has been removed as per the edits to the introduction.
11.	Pg 4 Sentence starting Line 17 would benefit from rewording- "Evidence shows that shared decision making regarding treatment and understanding between GPs and their patients is more pronounced and problematic in cross cultural consultations"	Thank you for your suggestion. We have amended as detailed across.	Page 3. We have edited this and it now reads, "Evidence shows that shared decision-making

			regarding treatment and understanding between GPs and their patients can be problematic in cross-cultural consultations.”
12.	References need to be checked for grammar, applying the et al. rule consistently, year provided where possible, completeness (for example 28)	We apologise for this oversight and have made great effort to ensure better documentation of the references.	See Reference section.
